# O Astronauta e a Jabuticaba

**DOI:** 10.36660/abc.20201098

**Published:** 2021-01-27

**Authors:** Andre d’Avila, Marco F. Vidal Melo

**Affiliations:** 1 Hospital SOS Cardio FlorianópolisSC Brasil Hospital SOS Cardio, Florianópolis, SC - Brasil; 2 Department of Anesthesia, Critical Care and Pain Medicine Massachusetts General Hospital BostonMassachusetts EUA Harvard Medical - Department of Anesthesia, Critical Care and Pain Medicine, Massachusetts General Hospital, Boston, Massachusetts – EUA

**Keywords:** Pandemia/prevenção e controle, Dexametasona, Anti-Inflamatórios, Hidroxicloroquina, COVID-19, SARS-CoV-2

A cada 2 anos, a partir de milhares de inscrições de todo o mundo, cerca de 100 pessoas, número semelhante ao de vagas anuais em muitas escolas médicas, são encaminhadas a NASA para uma serie de entrevistas, exames médicos, físicos e psicológicos. Assim como nas faculdades de medicina, o processo para selecionar astronautas é muito competitivo e tenta identificar os candidatos mais aptos às missões espaciais. O Conselho de Seleção procura por pessoas excepcionais dada a complexidade das tarefas a serem excetuadas e apenas 0,1% dos aspirantes a astronauta são selecionados. Tal como acontece com alunos de medicina, alguns candidatos cancelam sua inscrição após familiarizar-se com a tremenda carga de trabalho e perigos envolvidos em tornar-se um astronauta. Candidatos a médico e astronauta têm muito em comum: precisam ser pessoas motivadas, com enorme capacidade de concentração, fiéis ao método e ao treinamento exaustivo e capazes de compreender que equívocos em suas condutas podem ter consequências catastróficas.

Aos olhos dos pacientes, médicos serão sempre pessoas excepcionais tais como os astronautas. Quanto mais complexo o problema, melhor treinado e preparado o paciente espera que seu médico seja para poder lidar com aquela situação. Assim como na NASA, pacientes esperam que o médico por eles selecionados tenham sido excepcionalmente bem preparados e que possam tomar as melhores decisões pertinentes as suas vidas de maneira pragmática, informada e pronta. No lançamento do ônibus espacial, mesmo sabendo que tudo pode dar errado, não dá tempo de fazer figa nem torcer para que tudo dê certo: ou o astronauta está preparado ou não entra no foguete. Da mesma forma, a decisão do médico não pode se basear em crença ou esperança de que vai dar certo.

Diferentes tipos de pacientes optam por diferentes perfis de médico, mas todos, invariavelmente, buscam alguém que possa abordar o problema de acordo com condutas bem estabelecidas e predefinidas durante o treinamento e pela experiência do profissional. Nossos pacientes, e isso já foi estudado, preferem que seu destino dependa mais da técnica do que da sorte e indulgência. Entretanto, o controle de qualidade dos processos de formação do astronauta e do médico são muito diferentes. Nos primeiros, um grupo pequeno, o controle é mais fácil de ser exercido e os equívocos corrigidos com mais rapidez e objetividade. No caso dos médicos não. Menos durante na faculdade, mas principalmente depois de completado o treinamento formal, a qualidade e os resultados da formação ficam mais difíceis de ser avaliados. Os extremos são de reconhecimento relativamente simples: hospitais e profissionais de excelência *versus* imperícia ou negligência flagrantes. Mas existe uma enorme lacuna entre esses dois pontos onde, na verdade, a grande maioria dos atos médicos acontecem e o controle é mais complicado de ser exercido.

Isso ficou patente na pandemia.^1 , 2^ Entre o uso de ozônio por via retal - seria engraçado se não fosse trágico - e os estudos randomizados mostrando a ineficácia de varias intervenções, inúmeros médicos e instituições adotaram condutas à luz de sua experiência e convicção pessoal. Por vezes, inclusive, meramente convicção política, numa demonstração de desrespeito ao paciente transformado em cabo eleitoral durante a pandemia. Nesse contexto, para eximir-se de sua responsabilidade regulatória, *alguns* Conselhos de classe, com honrosas exceções, insistiram em que, dada a urgência, intervenções sem qualquer comprovação de eficácia fossem aceitas caso houvesse consenso entre o médico e o paciente. Curiosamente, acreditavam estar protegendo o médico com tal atitude. Mas se o consenso subjuga a regulamentação, e se não for possível impor ciência a impressões pessoais, para que Conselho? Em vez de insistir em promover o debate e a adoção de condutas baseadas em evidência científica, optaram pela estratégia de “se não fizer mal, pode”.

## Jabuticaba

O Brasil tem um ambiente médico peculiar. Intervenções tais como hidroxicloroquina, ivermectina desapareceram completamente do debate científico no resto do mundo. Essas e outras condutas não comprovadas, continuam a ser usadas apenas em ensaios clínicos. Em parte, graças a importantes contribuições brasileiras recentemente publicadas. Apesar de vários países terem aceito o resultado desses estudos brasileiros, conduzidos com todo o rigor que a gravidade da pandemia e o método cientifico exigem, muitos no Brasil insistem em questioná-los. Tal como a jabuticaba - natural e predominante, mas não exclusivamente nossa -, a discussão continua atual só no Brasil. Antes se dizia que os estudos feitos em outros países não se aplicavam à população brasileira. Agora, continuam a procurar o subgrupo do subgrupo onde a intervenção possa ser justificada e a partir dali extrapolar os eventuais resultados para toda a população mesmo sem qualquer comprovação científica. Como indivíduo, entendo que seja difícil aceitar evidência em contrário à convicção de alguém, mas como médico, essa atitude torna-se obrigatória.

Nesse ambiente de completa desregulamentação, vários colegas optaram por subscrever abaixo-assinado, criar sites promovendo condutas pessoais, escrever cartas a políticos e até organizar caravanas à Brasília para pressionar o Estado brasileiro a adotar condutas médicas por eles defendidas mesmo em face a evidência científica em contrário, ou seja, evidência da ineficácia da intervenção. Embora muitos deles sejam excelentes médicos em suas respectivas áreas de atuação, esse tipo de atitude só foi possível porque, com a desculpa de tentar ajudar, tudo se permite no Brasil. Houvesse algum tipo de norma propondo multa ou cassação da licença médica aos que promovessem condutas médicas não comprovadas, nada disso teria acontecido. Tal regulamentação seria, na verdade, semelhante a conduta dos conselhos éticos em casos de charlatanismo, quando atos médicos baseados em decisões consensuais não são aceitos como justificativa para isentar o infrator. Mas esse tipo de responsabilização e prestação de conta por condutas tomadas é muito falho ou inexistente no Brasil. Existe um termo em língua inglesa próprio para descrever essa imputabilidade chamada de *“accountability”* . Curiosamente, essa palavra não existe em português. Pra compensar, jabuticaba não tem tradução.

Este tipo de ato político, disfarçado de ato médico, congrega alguns pontos em comum. Primeiro, tenta criar um ambiente de insegurança junto a população e imprensa, sugerindo de maneira irresponsável que existe um conluio entre indústria farmacêutica, principais revistas médicas do mundo e pesquisadores para aprovar estratégias de alto custo e excluir populações menos privilegiadas. Essa desconfiança, frequente em momentos de crise, tornou-se quase certeza, quando dois trabalhos não-randomizados foram retirados de duas das mais importantes revistas médicas internacionais da atualidade. Mas ao contrário do que se apregoava, o sistema de avaliação da produção científica mostrou-se muito eficaz, crítico e resolutivo com essa ação rápida. Em apenas duas semanas, atento ao questionamento de outros médicos e investigadores, os trabalhos foram retirados pelos autores, a empresa que forneceu os dados desapareceu e o principal autor da publicação foi duramente repreendido pela escola médica onde trabalha. Não porque tenha agido premeditadamente com má-fé, mas porque não observou regras básicas de metodologia científica. O controle de qualidade funcionou desse lado da balança.

Mas os que se posicionam contra o método cientifico, atitude que tenta justificar a imobilidade e incapacidade de se organizar, continuam ilesos. Para o desalento deles, todavia, apenas a dexametasona foi confirmada como intervenção eficaz até o momento: uma droga barata, genérica e com licença para ser produzida em qualquer país do mundo sem pagamento de *royalty* . Mais uma vez, o complô não pode ser demonstrado.

Geralmente formado por pessoas sem nenhum interesse ou experiência em publicação científica, que encontram na resistência ao método uma maneira singular de colocarem-se em evidência sem nunca terem produzido nada, o grupo dos “descrentes” despendeu uma enorme energia nesse atual movimento anti-ciência no Brasil tentando convencer a todos sobre seus métodos e opiniões. Silenciosamente rejeitados pela maioria dos médicos, esses grupos continuam a buscar apoio fora do ambiente acadêmico, segundo eles, irremediavelmente corrompido. Ao invés disso, como fizeram outros, deveriam ter se organizado para responder a perguntas relevantes e beneficiar a população mundial como um todo. Mas isso dá trabalho: é sempre mais fácil resistir, reclamar e protestar do que produzir algo novo.

## Trabalhos Brasileiros

A pergunta mais importante no momento é: qual conjunto de três palavras tem feito mais vítimas? “Na minha experiência” ou “ensaio clínico randomizado”? A experiência, muitas vezes definida como a própria arte da medicina em si, é fundamental não para a interpretação dos dados de um estudo, mas para ajudar a acomodar aqueles resultados a um paciente e condição clínica específica. O estudo randomizado seleciona e afunila a análise sobre um grupo de pacientes para permitir a obtenção de resultados objetivos. A experiência permite a extrapolação e adaptação de resultados comprovados, mas não deve ser utilizada para interpretar resultados de pesquisa científica. Existem regras para isso e as diferenças de interpretação são também baseadas nessas normas e não em impressões pessoais.

Em contraponto aos grupos que defendem a experiência inédita e a memória recente como estratégias para a definição de condutas médicas, é preciso destacar o feito extraordinário de um grupo de pesquisadores brasileiros. Foram vários trabalhos publicados em revistas médicas importantíssimas no intervalo de algumas semanas. A iniciativa desses autores tem sido elogiada no mundo inteiro. Nenhum país conseguiu se organizar de maneira tão eficiente para responder questões relevantes à pandemia como o Brasil.

Só existe uma forma de aprendermos a tratar uma doença: pesquisa clínica. É um desafio tremendo. Uma pesquisa clínica bem-feita deve ser ampla, com muitos pacientes e cenários diferentes; ágil e coordenada, para resolver os problemas rapidamente e permitir avanços terapêuticos; e randomizada, com grupo controle para conseguir isolar o efeito do tratamento.^3^ Pouquíssimos países conseguiram coordenar movimentos dessa magnitude, que não só decifram a Covid-19 como nos fornecem respostas sobre o que fazer. O Brasil foi um deles. O grupo Coalizão, formado por hospitais líderes no país e que envolve mais de 50 centros nacionais, é hoje referência mundial. Serão cerca de 11 estudos para sanar dúvidas de como tratar a Covid-19. Graças ao Brasil, qualquer médico que queira tratar pacientes com Covid-19 aprendeu que a hidroxicloroquina, com ou sem azitromicina, não teve benefício em casos leves a moderados de Covid-19 (Coalizão I),^4^ e que a azitromicina também não teve benefício em casos graves (Coalizão II).^5^ Além de nos ensinar o que não funciona, o grupo ratificou que a Covid-19 grave tem tratamento: a dexametasona beneficiou pacientes hospitalizados com síndrome da angústia respiratória aguda (SARA) moderada-grave, acelerando a saída da ventilação mecânica (Coalizão III).^6^ Além dos estudos Coalizão, pesquisadores brazucas produziram estudos epidemiológicos de qualidade indiscutível, desenvolveram ensaios clínicos em condições precárias, sanaram a dúvida global sobre o que fazer com o paciente Covid-19 em uso de iECA ou BRA^7^ – sim, ele pode seguir com sua medicação –, e estão testando e produzindo vacinas com potencial de ajudar milhões de pessoas. Nos próximos 6 meses, são esperados novos estudos avaliando o efeito da hidroxicloroquina fora do ambiente hospitalar (tomara que funcione nesses pacientes como profilático), de diferentes anticoagulantes e do possível efeito antiviral do tociluzumabe dentre outros. Um feito incrível e sem precedentes no Brasil.

A pandemia mostrou o que há de melhor e pior na medicina brasileira. A politização de um conjunto de condutas médicas por médicos é inaceitável pois expõe a população aos riscos de tal conduta, criando factóides e crenças. Após o término da pandemia, os acertos parecerão naturais, óbvios e que sempre estiveram ali a nossa disposição. O esforço e a metodologia para a obtenção de tais respostas parecerão demasiados. As crenças e os achismos ficarão.

A ingestão de alho em casos de gripe é provavelmente uma das heranças da Peste Negra quando se acreditava que a transmissão da doença ocorria pelo mau odor – flegma – e o alho, que quando esmagado libera alicina, outras essências e perfumes poderiam prevenir a doença. Séculos depois, a crença popular ainda persiste: quem nunca tomou alho para tratar uma gripe?^8^ Inúmeros trabalhos sugerem que o alho tenha de fato efeito antiviral. Apesar de estudos randomizados nunca terem demonstrado tal benéfico de maneira contundente, o mito continua porque afinal de contas: 1) não custa nada tentar; mal não deve fazer; 2) se não cura pelo menos não atrapalha; 3) teve um amigo meu que tomou e ficou bom e 4) parece que funciona em outras doenças e 5) só estou tentando ajudar. Mais ou menos o mesmo nível de evidência que tem sido usado para justificar algumas intervenções na COIVD-19. Portanto, muitos podem legitimamente preferir tomar ivermectina e zinco em caso de gripe forte. Afinal, se havia uma suspeita, mesmo que infundada, de que funcionasse para a Covid-19, por que não funcionaria em uma gripe comum. Essa é uma das eventuais consequências nocivas de tais condutas instintivas não baseadas em estudos adequados.

E quem será responsabilizado por isso? Nessa hora, o movimento anti-ciência se divide entre os “convertidos”, os que desaparecem para mais tarde se mobilizar em torno de temas igualmente polêmicos e repetir a estratégia, e os que se organizam para tirar proveito financeiro da situação, continuando a prescrever e incentivando tais condutas. As consequências negativas para a saúde da população são incalculáveis. Portanto, não parece justo que apenas a resistência sem sentido tenha voz nesse momento. A população pode e deve confiar na ciência médica brasileira quando compreendida e exercida de maneira adequada. Houve um enorme amadurecimento da pesquisa clínica no Brasil por conta da pandemia. Muitos outros bons frutos virão a partir daqui. Ficou evidente nossa capacidade de aceitar e resolver desafios dessa magnitude. E quem sabe não está no extrato da jabuticaba a melhora da Covid-19!? Mas o que é realmente importante é a mensagem deixada pelos médicos e pesquisadores brasileiros às novas gerações: acreditem! Porque no final, o rigor e o treino do astronauta sempre prevalecem.
