# Supplementary material for: O Astronauta e a Jabuticaba
Source: Arq Bras Cardiol. 2021 Jan 27;116(1):1–3. [Article in Portuguese] doi: 10.36660/abc.20201098 (PMC8159492; doi:10.36660/abc.20201098)
Supplement: Translation

# Translation: The Astronaut and the Jabuticaba

**Authors:** Andre d’Avila, Marco F. Vidal Melo

**Affiliations:** 1 Brazil Hospital SOS Cardio, Florianópolis, SC - Brazil; 2 USA Harvard Medical School - Department of Anesthesia, Critical Care and Pain Medicine, Massachusetts General Hospital, Boston, Massachusetts – USA

**Keywords:** Pandemics/prevention and control, Dexamethasone, Anti-Inflamatory, Hydroxychloroquine, COVID-19, SARS-CoV-2

Every two years, of the thousands of applications from all over the world, only 100 are considered eligible to undergo medical, physical and psychological examinations at NASA for astronaut training. Similar to what happens in medical schools, the selection process to identify which candidates are qualified to fly on space missions is extremely competitive. Only 0.1% of applicants are accepted. Comparable to what happens with medical students, some of the candidates cancel their application once they become aware of the rigorous workload and risks of becoming an astronaut. Aspiring physicians and astronauts have similar traits – they need to be motivated, laser focused on tasks at hand, able to complete exhaustive training, and appreciate the possible catastrophic consequences associated with misconduct.

Physicians, like astronauts, are frequently perceived as exceptional individuals who are capable of making pragmatic and prompt decisions based on the best available information. Medical decision making, like a shuttle launch, requires thorough preparation rather than blind faith that with keeping one’s fingers crossed everything will be ok. Patients look to a physician who can make informed decisions coupling evidence-based medicine, guidelines and professional experience. However, variations in clinical practice are common. While it is easy to separate the extremes of excellent care from flagrant malpractice it remains a large gap between these two boundaries, where medical decisions are often made and adequate quality control is difficult.

This lack of oversight in “grey”^1 , 2^zones has become clear during the COVID-19 pandemic. From rectal ozone therapy – funny, if it were not tragic – to studies showing the inefficacy of several therapies, many physicians and institutions have made therapeutic decisions based on anecdotal experience or personal belief and, not rarely, on political conviction. In this context, to exempt themselves from their regulatory responsibilities, *some* medical councils, with honorable exceptions, have advocated that interventions without proven efficacy could be accepted if consensus between the doctor and the patient exists. However, if unanimity subjugates legislation, and if science cannot prevail over personal impressions, what is the value of such councils? Instead of promoting fruitful debates and adoption of evidence-based practice, a strategy of “if it is not bad, why not?” became acceptable.

## Jabuticaba

Brazil has a peculiar medical environment. Although interventions like hydroxychloroquine and ivermectin disappeared from the international scientific debate once disproved in clinical trials, Brazilian practitioners continue to debate the validity of these studies. Like *jabuticaba* – a fruit native to and predominant (but not exclusive) in Brazil – this debate is still current only in this country. Previous attempts to discount the results of studies with the argument that they had been conducted abroad and could not be extended to the Brazilian population can no longer be justified as many of these studies have included Brazilian patients. As individuals, we do understand the difficulty in accepting evidence opposing someone’s conviction; however, as a doctor, this attitude is indefensible.

In this context of conviction and belief over evidence and data, several practitioners have created websites and even solicited the government to support and disseminate their practice despite proven inefficacy of the proposed interventions. Even though many are excellent physicians in their fields, this situation has only been possible because, under the auspices of trying to help, almost anything has been allowed in Brazil. If there were any regulation proposing a fine or termination of medical license to those who supported unproven medical practices, none of this would have happened. This regulation would be, in fact, similar to previous decisions of ethics committees in cases of charlatanism, when medical practices based on consensual decisions are not accepted as justification to exempt the infringer. However, these types of regulations are defective or absent in Brazil. Curiously, the term “accountability”, that in English means an obligation or willingness to accept responsibility for one’s actions, does not exist in Portuguese. On the other hand, there is no English word for “ *jabuticaba* ”.

Those promoting unproven medical practices are likely unknowingly participating in the political non-sense debate surrounding the COVID-19 pandemic. Their engagement creates an unsafe atmosphere around both population and the press, as they irresponsibly suggest an alliance between the pharmaceutical industry, important medical scientific journals, and researchers to approve high-cost strategies and exclude less privileged populations. This conspiracy theory, common in situations of crisis, became almost a certainty when two non-randomized trials were published in two of the most important international medical journals today. But the scientific peer-review process was shown to be very effective, critical and resolute in response to the concerns from other independent physicians and researchers. In only two weeks, the studies were retracted by the authors and the company that had provided the data disappeared. Furthermore, the main authors were sharply rebuked by the medical schools where they work, because in an effort to help, they disregarded the basic principles of scientific methodology. This is to be contrasted with the fact that Brazilian physicians who have stood against science suffer no consequence. These groups have spent enormous energy on the anti- science movement in Brazil, trying to convince the general population about their opinions. Silently rejected by most physicians, the anti-science movement has gained traction outside the academic realm, the latter which they deem as irretrievably corrupted. Instead, they should have positioned themselves to help answering important questions to benefit the whole population. However this is a lot of work! It is always easier to resist, complain and protest than to produce something scientifically relevant.

## Brazilian Studies

Which three words have created more victims, “in my opinion” or “randomized clinical trials”? Although experience, or the “art of medicine”, is valuable, it should complement the interpretation of scientific data and help apply results of scientific studies to specific patients and clinical situations. This contrasts with the belief that the “art of medicine” is simply a tool to promote one’s anecdotal experience and recent memory of medical practice as strategies to establish standards of care. This approach devalues the extraordinary work of Brazilian researchers, who in a few weeks published several papers in renowned medical journals receiving global recognition. Their work set Brazil apart from other nations who have not been able to scientifically answer as many important questions about the COVID-19 pandemic as the Brazilian medical community.

The only way to advance medical practice is through well done clinical investigation. Few countries have been able to coordinate the processes necessary to perform well done, impeccable investigation to answer the challenges of COVID-19. Brazil is one of the countries that was up to it. Today, the *Coalition* group, consisting of leading hospitals and more than 50 national centers, is a global reference. Nearly 11 studies have been conducted on COVID-19 treatment.^3^Thanks to Brazil, physicians have learned that hydroxychloroquine with or without azithromycin did not improve clinical status of patients with mild-to-moderate COVID-19 (COALITION I),^4^and that azithromycin is not effective in severe COVID-19 also (COALITION II).^5^In addition to teaching us what not to do, the group has also confirmed that severe COVID-19 can be treated: hospitalized patients with moderate or severe acute respiratory distress syndrome (ARDS) due to COVID-19 benefited from intravenous dexamethasone, increasing the number of ventilator-free days (COALITION III).^6^Besides the COALITION studies, Brazilian researchers have produced high-quality epidemiological work, developed clinical trials in precarious conditions, answered the global question of how to treat COVID-19 patients receiving angiotensin-converting enzyme inhibitors and angiotensin receptor blockers^7^– yes, these therapies may be continued – and have been testing and producing vaccines that will potentially help millions of people. In the next six months, new studies will evaluate the efficacy of hydroxychloroquine in the out-of-hospital setting (hopefully it has a prophylactic effect), the role of different anticoagulants and the antiviral effect of tociluzumab. This is an astonishing and unprecedented achievement for Brazil.

The COVID-19 pandemic has revealed the best and worst of Brazilian medicine. The unscientific approach to medicine is unacceptable, places the population at risk, creates fake-news, and overshadows excellence in scientific endeavors within our country. After the pandemic, our success will seem natural, obvious, and inevitable; the efforts and methods to obtain all the answers will seem excessive; but dogmas will remain.

The use of garlic to treat patients with influenza is likely a remnant of the Black Death, when it was believed that transmission of the disease occurred through bad odors – phlegm -, and garlic and other essences could prevent the disease. Centuries later, this popular belief persists. Who has never eaten garlic to treat a flu?^8^Numerous studies have suggested that garlic has an antiviral effect. Although there are no randomized studies showing these effects, the myth still exists, since: 1) “it is probably not bad, so why not?”; 2) “it may not cure you, but it could help you”; 3) “a friend of mine used it and got better”; 4) “it seems to work in other diseases”; 5) “I am just trying to help”. These are almost the same level of evidence on which some interventions in COVID-19 have been based. In the future, many patients could genuinely prefer to take ivermectin or zinc in case of severe influenza, since “if it could be effective for COVID-19, why would it not be effective for a common flu?” The trickle-down effect of unvalidated practices is not trivial.

The anti-science movement is currently divided among 3 groups: 1) the “converted” ones, 2) those who will be off the scene and show up again using the same strategy when another pandemic issue arises, and 3) those who take financial advantage of the situation by prescribing these drugs of unknown efficacy and encouraging these practices. Consequences to population health can be disastrous. Thus, it does not seem fair that only those who refuse to place their trust in the scientific method have a say today. Clinical research in Brazil has evolved greatly during the pandemic: it has become clear that the scientific community within our country has the capacity to overcome colossal challenges. The population may and should trust Brazilian medical science when properly understood and applied. And who knows, perhaps the key element to treat COVID-19 will be found in the *jabuticaba* extract?! What really matters is the message to the new generation of Brazilian doctors and researchers: just like for astronauts, rigor and training always prevail in the end.
